# AI-Generated Exercise Prescriptions for At-Risk Populations: Safety and Feasibility of a Large Language Model Assessed by Expert Evaluation

**DOI:** 10.3390/jcm15062457

**Published:** 2026-03-23

**Authors:** Minkyung Choi, Jaeyong Park, Myeounggon Lee, Jaewon Beom, Se Young Jung, Kihyuk Lee

**Affiliations:** 1 Department of Sports Culture, Dongguk University, Seoul 04620, Republic of Korea; 2 Department of Fitness Rehabilitation, Sun Moon University, Asan 31460, Republic of Korea; 3 Institute on Aging, Seoul National University, Seoul 08826, Republic of Korea; myeounggon.lee@gmail.com; 4 Department of Rehabilitation Medicine, Seoul National University Bundang Hospital, Seongnam 13620, Republic of Korea; rehabbjw@gmail.com; 5 Department of Family Medicine, Seoul National University Bundang Hospital, Seongnam 13620, Republic of Korea; 6 Department of Family Medicine, College of Medicine, Seoul National University, Seoul 03080, Republic of Korea; 7 Office of Hospital Information, Seoul National University Bundang Hospital, Seongnam 13605, Republic of Korea

**Keywords:** generative artificial intelligence, exercise prescription, expert evaluation, prompt engineering, large language models

## Abstract

**Background/Objectives:** In exercise science and sports medicine, the potential use of large language models for generating personalized exercise programs is being explored. However, the practical applicability of AI-generated exercise prescriptions has not yet been sufficiently validated, particularly in complex clinical contexts. This study aimed to evaluate their practical utility under expert supervision. **Methods:** Exercise prescription outputs generated by a large language model (Gemini 2.5, Google LLC) were analyzed using clinical cases incorporating complex exercise-related considerations. Three levels of prompt structuring were applied. Experts evaluated the outputs using a structured rubric assessing safety, feasibility, guideline alignment, and personalization. Inter-expert agreement was assessed using intraclass correlation coefficients (ICC), and expert-specific internal consistency was evaluated using Cronbach’s alpha. **Results:** AI-generated exercise prescriptions demonstrated a certain level of structural completeness. However, inter-expert agreement was low (ICC (2,3) = 0.139), whereas expert-specific internal consistency was high (Cronbach’s alpha > 0.92). Prompt structuring from Stage 1 to Stage 2 was associated with improved mean scores in safety and guideline alignment. Additional structuring did not consistently yield further improvements. **Conclusions:** AI-generated exercise prescriptions may have practical potential as supportive decision-making tools when expert involvement is assumed. Nonetheless, expert judgments did not converge toward a single evaluative standard, reflecting the inherently expert-dependent nature of exercise prescription.

## 1. Introduction

Recent advances in generative artificial intelligence (Generative AI), particularly large language models (LLMs), have introduced new possibilities across the fields of healthcare and health management [1,2]. These models are capable of generating contextually appropriate text based on user prompts and have been discussed as potentially applicable tools in various domains, including clinical consultation support, health information delivery, facilitation of behavior change, and personalized exercise advice [3,4,5]. In particular, within the fields of exercise science and sports medicine, LLMs have been suggested as tools that could assist in providing exercise guidance for non-expert users or support the development of individualized exercise programs [6,7].

However, exercise prescription is fundamentally distinct from the simple provision of exercise-related information [8,9]. Exercise prescription requires comprehensive consideration of an individual’s health status, disease characteristics, functional capacity, medication use, exercise contraindications, and precautionary criteria. This is especially critical for individuals with chronic conditions such as hypertension, diabetes, musculoskeletal disorders, and cardiovascular risk factors, for whom careful program design is necessary to minimize the risk of adverse events or exercise-related complications [10,11]. Owing to these characteristics, exercise prescription remains largely expert-driven, and the application of automated systems or artificial intelligence-based approaches necessitates sufficient validation and cautious implementation [12,13].

Recent studies have reported the use of LLMs, including ChatGPT, to generate resistance training programs, followed by evaluations conducted by exercise science experts based on established scientific guidelines [14]. In addition, studies in which coaching professionals directly evaluated running training programs generated by LLMs have shown that, although the generated plans exhibited a certain level of structure and internal logic, they did not reach an “optimal” level from an expert perspective [15]. Taken together, these findings suggest that LLMs demonstrate potential to generate structured exercise programs that reflect basic exercise science principles; however, recurring limitations have been identified with respect to the quantitative prescription of exercise intensity, consistency in the application of progressive overload, and the extent to which individual characteristics are adequately reflected [14,15].

Other studies have reported that increasing the amount and specificity of information included in prompts leads to improvements in the quality of generated exercise programs, suggesting that LLM-based exercise prescription outcomes are highly dependent on user input [15,16]. In a study involving individuals with type 2 diabetes mellitus, expert-blinded evaluations of exercise programs generated by ChatGPT revealed that while some plans aligned with clinical guidelines, many failed to sufficiently account for disease-specific characteristics and contraindications [17]. These findings indicate that, despite their potential, LLM-based exercise prescriptions continue to present important limitations with respect to safety and reliability [16,18].

Meanwhile, previous studies have primarily evaluated the quality of LLM-generated exercise programs under relatively simple or constrained conditions, often focusing on healthy individuals or single-disease populations. As a result, empirical validation involving individuals with complex and overlapping health risk factors, who are most commonly encountered in real-world clinical and exercise settings, remains limited [19,20,21]. Furthermore, the complexity and risk associated with exercise prescription increase substantially when multiple diseases or functional limitations coexist, compared with single-disease conditions [22,23]. In such complex contexts, expert evaluations of the same exercise prescription output are more likely to vary depending on interpretive perspective. From this standpoint, examining the practical applicability and limitations of exercise programs generated by generative AI is of critical importance for determining the real-world feasibility of LLM-based exercise prescription [24,25].

Therefore, the purpose of this study was to analyze exercise prescription outputs generated by a generative AI based on clinical cases incorporating complex exercise-related considerations through expert evaluation and to examine the extent to which these outputs can be practically utilized in clinical and exercise settings when expert involvement is assumed. Accordingly, this study aims to provide foundational evidence for future research and practical applications of generative AI-based exercise prescription by presenting both the potential applicability of LLM-based exercise prescription and the limitations and considerations that should be addressed prior to real-world implementation.

## 2. Materials and Methods

### 2.1. Study Design

**Figure 1:**
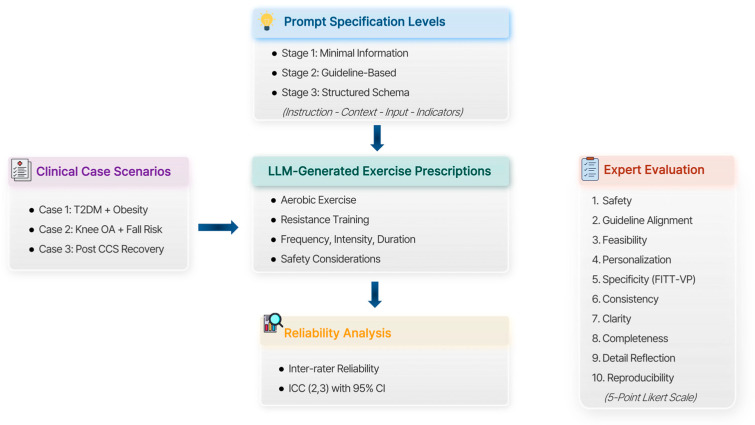
Study design and expert evaluation framework. T2DM, type 2 diabetes mellitus; Knee OA, knee osteoarthritis; Post-CCR recovery, post-colon cancer resection recovery; FITT-VP, frequency, intensity, time, type, volume, progression.

This study is an expert-based evaluation study targeting exercise prescription outputs generated by a large language model (LLM). Rather than assessing the accuracy or predictive performance of AI-generated exercise plans or comparing models, the study focuses on the qualitative level at which exercise prescriptions are evaluated in complex and high-risk exercise situations. Accordingly, the AI-generated exercise prescriptions themselves were set as the evaluation target in a descriptive evaluation study examining the consistency and characteristics of expert judgments. A schematic overview of the study design is presented in Figure 1.

### 2.2. Clinical Case Construction

Three fictional clinical cases were constructed in which two or more exercise-related considerations coexist simultaneously (Table 1): (1) type 2 diabetes mellitus with obesity, (2) knee osteoarthritis with fall risk, and (3) recovery after colon cancer surgery. Clinical cases were selected through prior consultation between the research team and experts in clinical and exercise prescription fields based on the following criteria: (1) clearly defined exercise contraindications or precautionary considerations, (2) the need to integrate aerobic, resistance, and functional exercise components, and (3) representation of high-risk exercise prescription contexts across metabolic, musculoskeletal, and oncological domains. Fictional cases were used to avoid ethical concerns related to real patients and to allow systematic control of clinical complexity. The clinical cases were designed to reflect realistic high-risk exercise prescription contexts involving multimorbidity, incorporating key elements considered in practice, such as age, sex, disease characteristics, functional limitations, and exercise-related precautions. Sex was included as part of the clinical profile in each case but was not treated as an analytical variable. The full textual descriptions of the clinical cases used as input prompts are provided in Supplementary Material S1.

### 2.3. Prompt Design and Exercise Plan Generation

For each clinical case, three stages of prompts were applied (Supplementary Material S1). First, the Minimal Information Prompt consisted of a simple prompt including only minimal basic information. Second, the Guideline-Based Prompt represented an intermediate-level prompt incorporating international exercise guidelines and contraindications, including those from the American College of Sports Medicine (ACSM), the American Diabetes Association (ADA), and the Osteoarthritis Research Society International (OARSI). Third, the Structured Schema (Four-Component Format) Prompt applied an advanced prompt engineering approach composed of Instruction, Context, Input Data, and Output Indicator, with standardized output formats for safety-related elements (Safety Box) and personalization elements (Personalization Box). Detailed descriptions of each component and output format are provided in Supplementary Material S1. The prompts were designed not to optimize model performance but to reflect differences in user input that may occur in real-world settings. The order of prompt conditions was varied across sessions to reduce potential sequencing effects.

### 2.4. AI Model and Session Control

All exercise prescription outputs used in this study were generated using Google Gemini 2.5 (Google LLC, Mountain View, CA, USA), which was commercially available at the time of the experiment (early November 2025). To exclude potential interference from model personalization, prior conversation history, or accumulated contextual information, all prompts were entered in independent new guest sessions, and each session was reset before generation. All interactions were conducted in non-logged-in guest sessions using the platform’s default system settings. Because the model was accessed via the public web interface rather than the API, configuration parameters such as temperature and top-p were not user-adjustable and remained at their default values. No explicit reasoning strategies, such as Chain-of-Thought (CoT) prompting, were employed. In addition, no explicit output-length constraints were applied. This approach was intended to evaluate the model’s baseline generative performance under standardized conditions.

Considering the non-deterministic nature of LLMs, exercise plans were generated five times under identical clinical case and prompt conditions to evaluate response consistency within the same conditions. This repetition was not intended to optimize model performance but to assess the consistency of generated outputs under identical conditions. Given three clinical cases and three prompt stages, this process resulted in a total of 45 original outputs (3 cases × 3 prompt stages × 5 repetitions). No researcher-driven editing or modification was applied to the generated content. Representative examples of AI-generated exercise prescriptions across prompt stages are provided in Supplementary Material S2.

### 2.5. Evaluators, Evaluation Criteria, and Evaluation Procedure

Three experts holding PhD degrees in sports medicine or clinical exercise science, with substantial experience in exercise prescription for individuals with multimorbidity and high-risk conditions, independently evaluated the exercise prescriptions. Evaluators were informed that the plans were AI-generated in order to encourage careful clinical scrutiny; however, they remained blinded to the prompt stage and repetition conditions to minimize potential bias. To further reduce sequence-related bias and evaluator fatigue, all 45 generated plans (3 cases × 3 stages × 5 repetitions) were presented to each expert in a fully randomized order. Furthermore, the evaluation was not conducted in a single session; instead, it was distributed across multiple sessions over several days to maintain evaluators’ attention and scoring consistency. Exercise plans were assessed using a structured 10-item evaluation rubric developed through research team discussion based on established exercise prescription principles, international guidelines, prior literature, and expert consensus. The domains included safety, guideline alignment, feasibility, personalization, FITT-VP (frequency, intensity, time, type, volume, progression) specificity, logical consistency, clarity, completeness, reflection of condition-specific considerations, and consistency across repetitions. Each item was rated on a 5-point Likert scale, and an overall score was calculated as the mean of the ten items. Detailed scoring definitions are provided in Supplementary Material S3. Before formal evaluation, a brief calibration session was conducted to align the interpretation of rubric items without modifying scoring criteria. Evaluations were performed independently. When information was unclear or insufficient, evaluators applied a conservative scoring approach, prioritizing safety. To systematically interpret the observed scoring variability, a thematic analysis was conducted on the qualitative feedback provided by the evaluators (Supplementary Material S4). Following the six-step framework proposed by Braun and Clarke [26], two researchers independently coded the free-text comments to identify recurring themes related to the strengths and limitations of each prompt stage. This qualitative analysis complemented the quantitative findings by providing additional insight into how experts interpreted safety and feasibility across different prompt conditions.

### 2.6. Statistical Analysis

Descriptive statistics, including means and standard deviations, were calculated for each evaluation item and the overall score. Although evaluation scores are ordinal in nature, mean values were used at the descriptive level to facilitate comparison across prompt conditions and to examine overall trends. As this study represents an evaluation study rather than a comparison of predictive models or performance optimization, the analysis focused specifically on the quality characteristics of AI-generated exercise prescriptions and the consistency of expert evaluations. To ensure statistical validity, each generated exercise plan, including repeated generations under identical prompt conditions, was treated as an independent evaluation unit. This approach was justified by the fact that outputs were generated across separate sessions without shared contextual memory, rendering each iteration an independent realization of the input condition. Inter-expert reliability was assessed using the intraclass correlation coefficient (ICC) based on a two-way random-effects model with an absolute agreement definition for the average measures of three experts (ICC (2,3)). Additionally, the internal consistency of the 10-item evaluation rubric for each evaluator was examined using Cronbach’s α. All statistical analyses were conducted using IBM SPSS Statistics (version 25; IBM Corp., Armonk, NY, USA). To visualize score distributions and evaluator trends, box-and-whisker plots with jittered data points were generated using Python 3.12 (with Matplotlib 3.8.2 and Seaborn 0.13.2 libraries). Generative AI tools were used solely for language editing and proofreading.

## 3. Results

### 3.1. Expert-Specific and Overall Mean Scores by Prompt Stage and Clinical Case

Table 2 presents expert-specific and overall mean scores of AI-generated exercise prescriptions across three prompt stages for each clinical case. Across all cases, overall mean scores tended to increase from Stage 1 to Stage 2 (e.g., from 3.33 to 3.63 in the type 2 diabetes with obesity case and from 3.61 to 3.81 in the knee osteoarthritis with fall risk case), whereas changes from Stage 2 to Stage 3 were inconsistent across cases. For the type 2 diabetes with obesity case, the overall mean score increased across prompt stages, reaching the highest value at Stage 3 (3.91). In contrast, for the knee osteoarthritis with fall risk case, the overall mean score increased from Stage 1 (3.61) to Stage 2 (3.81) but showed no further improvement at Stage 3 (3.76). For the post-colon cancer surgery recovery case, the overall mean score increased slightly at Stage 2 (3.77) and decreased at Stage 3 (3.37). At the expert level, responses to increased prompt specificity varied across experts and cases, resulting in heterogeneous scoring patterns, particularly at the highest prompt specificity level.

### 3.2. Inter-Expert Reliability and Internal Consistency of Expert Evaluations

Table 3, Table 4 and Table 5 summarize inter-expert reliability and internal consistency of expert evaluations. Overall inter-expert reliability for the total score was low (ICC (2,3) = 0.139, 95% CI: −0.350 to 0.482; Table 3). Expert-specific internal consistency across the 10 evaluation items was high, with Cronbach’s α values ranging from 0.923 to 0.943 across experts (Table 4).

**Figure 2:**
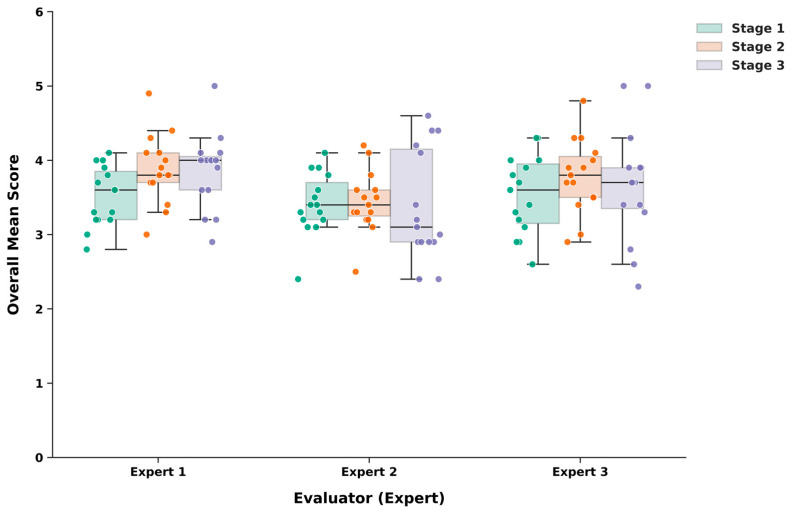
Distribution of mean evaluation scores by expert across prompt stages. Individual data points (*n* = 135) represent five repeated iterations for each clinical case. The plots illustrate individual evaluator trends and scoring variances despite differences in baseline ratings.

As illustrated in Figure 2, the individual scoring distributions clarify the discrepancy between low inter-expert reliability and high internal consistency. While Expert 2 consistently assigned lower scores across all stages compared to Expert 1 and Expert 3 (explaining the low ICC), all three evaluators demonstrated a synchronized upward trend from Stage 1 to Stage 2.

At the item level, inter-expert reliability varied across evaluation domains (Table 5). Positive ICC values were observed for items such as Clarity (ICC (2,3) = 0.384, 95% CI: 0.015 to 0.635) and Safety (ICC (2,3) = 0.201), whereas negative ICC values were observed for Guideline Alignment (ICC (2,3) = −0.358) and Specificity (FITT-VP) (ICC (2,3) = −0.432).

### 3.3. Item-Level Mean Score Comparison Across Prompt Specificity Levels

Table 6 presents item-level mean scores across three prompt specificity levels based on averaged expert ratings. Across evaluation items, mean scores generally increased from Stage 1 to Stage 2, whereas changes from Stage 2 to Stage 3 were item-dependent. Several items, including Safety (3.69 ⟶ 4.07 ⟶ 3.69) and Guideline Alignment (3.80 ⟶ 4.16 ⟶ 3.98), showed higher mean scores at Stage 2 compared with Stage 1, followed by stable or slightly lower scores at Stage 3. In contrast, items such as Clarity (3.51 ⟶ 3.76 ⟶ 3.78) and Completeness (3.58 ⟶ 3.73 ⟶ 3.93) showed incremental increases across prompt stages. Other items, including Feasibility, Personalization, and Reproducibility, exhibited relatively modest changes across stages. Median and interquartile range (IQR) values for each evaluation item are additionally reported in Supplementary Material S5.

## 4. Discussion

Exercise prescription is a decision-making domain that presupposes expert clinical judgment, and in this context, the present study analyzed the evaluative characteristics of exercise prescription outputs generated by generative AI through expert evaluation. The results of this study demonstrate that, although AI-generated exercise prescriptions exhibit a certain level of structure and formal completeness, expert evaluations do not converge on a single judgment standard. Accordingly, this discussion examines the evaluative characteristics and practical implications of AI-generated exercise prescription outputs based on expert evaluation results.

The concurrent observation of low inter-expert agreement (ICC) and high expert-specific internal consistency (Cronbach’s α) in this study can be interpreted not as contradictory findings but rather as reflecting the structural characteristics inherent in expert evaluation. In reliability research, ICC and Cronbach’s α capture conceptually distinct aspects of measurement, and discrepancies between the two indices do not necessarily indicate methodological inconsistency but rather reflect differences between agreement and internal coherence constructs [27]. Cronbach’s α indicates the extent to which a single expert applies evaluation criteria consistently across multiple items, whereas ICC reflects the degree to which different experts provide similar judgments for the same target. The consistently high Cronbach’s α values observed across experts (all exceeding 0.92) suggest that the evaluation rubric functioned reliably within each expert’s individual judgment framework. In other words, each expert interpreted and evaluated the AI-generated exercise prescriptions in a relatively consistent manner according to their own criteria, making it unlikely that the evaluation process was arbitrary or disorganized.

In contrast, inter-expert agreement based on overall scores was low (ICC (2,3) = 0.139), and agreement at the item level was also limited, with the exception of a small number of items. Negative ICC values observed in some items may partly reflect sampling variation, particularly given the small number of raters and the variability in expert evaluative criteria. This finding may suggest that experts may have applied differing interpretive criteria and item-level weighting schemes when evaluating the same exercise prescription outputs. A follow-up thematic analysis based on the Braun and Clarke framework further explored this discrepancy and revealed divergent clinical priorities among experts [25]. While some evaluators prioritized strict adherence to safety contraindications, others placed greater emphasis on practical feasibility and the progression of exercise intensity (Supplementary Material S4). Such results can be interpreted as reflecting the fact that exercise prescription often involves context-sensitive clinical reasoning, in which multiple acceptable approaches may coexist depending on clinical context, risk perception, and practical experience [8,9,10,11,23]. Variability among experts has been widely documented in clinical decision-making research, particularly in domains characterized by multimorbidity and uncertainty, where multiple defensible decisions may coexist [23,28].

Previous studies on AI-based exercise prescription have primarily evaluated the appropriateness of generated prescriptions using metrics such as mean scores, safety, and guideline adherence, and even when expert evaluation was included, results were often summarized using a single mean value or a single reliability index. In contrast, the present study simultaneously analyzed inter-expert agreement and expert-specific internal consistency, thereby structurally demonstrating that AI-generated exercise prescriptions can be evaluated consistently within individual experts yet do not converge toward a single expert standard. This finding suggests that the complexity of expert judgment in generative AI-based exercise prescription may not be fully captured using simple mean scores or a single reliability metric alone.

The observed increase in mean scores from Stage 1 to Stage 2 across multiple evaluation items suggests that prompt structuring may have contributed to differences in expert evaluation scores. Specifically, the mean Safety score increased from 3.69 in Stage 1 to 4.07 in Stage 2, and the Guideline Alignment score increased from 3.80 to 4.16. These descriptive differences suggest that when guideline-based conditions and evaluation criteria are explicitly incorporated into prompts, AI-generated exercise prescriptions may become more closely aligned with expert evaluation standards [15,17]. Because the number of expert raters was limited and the primary aim of this study was exploratory evaluation of LLM-generated exercise prescriptions, formal statistical comparisons between prompt stages were not performed.

Previous studies have likewise reported that exercise programs generated under prompts explicitly specifying guidelines and safety conditions received relatively higher expert evaluation scores. These findings suggest that prompt structuring may not enhance the AI’s intrinsic judgment capability per se but rather function to align outputs with formats and criteria that are more readily evaluable by experts. Accordingly, the score increases observed at Stage 2 in the present study are more plausibly interpreted as reflecting reduced misalignment between evaluation criteria and output structure, rather than an improvement in the AI’s underlying decision-making ability.

By contrast, at Stage 3, consistent additional score improvements were not observed across multiple items (Table 5). For example, the mean Safety score increased from 3.69 in Stage 1 to 4.07 in Stage 2 but remained at 3.69 in Stage 3, while Guideline Alignment peaked at Stage 2 and showed a slight decrease at Stage 3. These findings suggest that the effects of prompt structuring may operate in a nonlinear manner beyond a certain threshold and that additional formalization beyond guideline-based structuring did not consistently translate into higher expert scores. Prior work in prompt engineering has similarly suggested that increasing instruction specificity does not uniformly improve output quality and that over-constrained prompts may limit adaptive reasoning or contextual flexibility [13,29]. This plateau in performance may reflect a limitation of general-purpose LLMs when operating under highly rigid and standardized prompt structures. Such structured prompts were intentionally designed by clinical experts to reflect guideline-based constraints in exercise prescription. While further technical optimization of prompts may be possible, this study prioritized maintaining methodological comparability by applying a consistent prompt architecture across all clinical cases. In other words, as constraints become increasingly detailed, important elements such as individual clinical context and acceptable risk tolerance that are critical to expert decision-making may not be adequately expressed in the generated outputs [15,17].

Taken together, these findings indicate that prompt structuring can contribute to the evaluability of AI-generated exercise prescriptions; however, beyond a certain point, it does not continuously enhance the level of practical optimality required by experts. This suggests that generative AI-based exercise prescription may be positioned as a structured decision-support tool that assists expert clinical judgment rather than replacing it [1,2,18].

The present study has several limitations. First, the number of expert evaluators was limited, and as a result, the sample size may have been insufficient to allow for stable estimation of inter-expert agreement (ICC). As reliability coefficients such as ICC may become unstable when the number of raters is small, the agreement estimates reported in this study should be interpreted with caution. Second, this study did not include a human-generated gold-standard control group for direct comparison. While our focus was on the baseline performance of the LLM itself, future studies should employ blinded comparisons between AI-generated and human-generated exercise prescriptions to further validate these findings. Third, the evaluation rubric was specifically developed for the purposes of this study based on existing exercise prescription guidelines and expert discussion, and formal validation of the rubric was not conducted. Therefore, caution is required when generalizing the findings to other clinical contexts. In addition, multiple outputs were generated under identical prompt and case conditions to examine the consistency of LLM responses. Because these outputs originate from the same prompt context, they may not be fully statistically independent, and the findings should therefore be interpreted as exploratory observations of response variability. Lastly, as this study focused on evaluating the baseline capabilities of a general-purpose LLM via zero-shot prompting, future research should integrate domain-specific knowledge retrieval systems, such as Retrieval-Augmented Generation (RAG), to further enhance clinical safety. Future studies should expand the number of expert evaluators and conduct subgroup analyses by expert type to more precisely explore differences in agreement structures. Despite these limitations, the present study is meaningful in that it explicitly delineates the evaluation structure and interpretive framework of generative AI-based exercise prescription, thereby serving as foundational evidence for future research and practical applications.

## 5. Conclusions

This study analyzed exercise prescription outputs generated by generative AI from the perspective of expert evaluation, examining differences in evaluative characteristics and expert judgment according to the level of prompt structuring. The results indicate that generative AI-based exercise prescriptions were able to achieve evaluability and a minimal level of quality through a certain degree of structuring; however, expert judgments did not converge toward a single standard. A formal thematic analysis revealed that this low agreement was driven by divergent clinical priorities, such as the trade-off between strict safety and practical progression. These findings suggest that AI-generated exercise prescriptions have practical potential as a supportive decision-making tool rather than a substitute for clinical judgment, particularly for high-risk populations where expert involvement and professional verification remain essential.

## Figures and Tables

**Table 1:** Characteristics of the hypothetical clinical cases used for AI-generated exercise prescription.

Clinical Cases	Sex	Age (Years)	Primary Condition(s)	Key Functional Limitation or Risk	Baseline Physical Activity Level	Primary Exercise Goal
Clinical Case 1 (Type 2 diabetes + obesity)	Male	55	Type 2 diabetes mellitus, obesity	Limited exercise experience, mild peripheral neuropathy	Low	Weight reduction and improved glycemic control
Clinical Case 2 (Knee osteoarthritis + fall risk)	Female	70	Knee osteoarthritis	Knee pain during walking, prior fall incident	Low	Pain reduction, maintenance of walking ability, and fall prevention
Clinical Case 3 (Post-colon cancer surgery recovery)	Male	60	Post-colon cancer surgery	Deconditioning, limited walking endurance, fatigue	Low	Physical recovery, fatigue reduction, and improvement of lifestyle habits

All clinical cases were fictional and developed for research purposes. Baseline physical activity level was qualitatively defined based on clinical case description and not measured using a standardized instrument.

**Table 2:** Expert-specific and overall mean scores of AI-generated exercise prescriptions by prompt stage and clinical case.

Clinical Case	Prompt Stage	Expert 1 Mean Score	Expert 2 Mean Score	Expert 3 Mean Score	Overall Mean Score
Type 2 diabetes + obesity	Stage 1	3.38 ± 0.49	3.30 ± 0.76	3.30 ± 0.81	3.33 ± 0.70
	Stage 2	3.82 ± 0.69	3.38 ± 0.73	3.68 ± 0.74	3.63 ± 0.74
	Stage 3	3.78 ± 0.68	4.34 ± 0.52	3.62 ± 0.88	3.91 ± 0.77
Knee osteoarthritis + fall risk	Stage 1	3.56 ± 0.70	3.44± 0.70	3.84 ± 0.65	3.61 ± 0.70
	Stage 2	4.12 ± 0.63	3.54 ± 0.58	3.78 ± 0.71	3.81 ± 0.68
	Stage 3	4.20 ± 0.40	2.96 ± 0.67	4.12 ± 0.82	3.76 ± 0.86
Post-colon cancer surgery recovery	Stage 1	3.60 ± 0.57	3.50 ± 0.51	3.46 ± 0.76	3.65 ± 0.69
	Stage 2	3.70 ± 0.58	3.40 ± 0.49	3.90 ± 0.81	3.77 ± 0.71
	Stage 3	3.60 ± 0.64	2.86 ± 0.64	3.24 ± 0.74	3.37 ± 0.82

Values are presented as mean ± standard deviation (SD). Expert-specific mean scores were calculated across five repeated generations under the same prompt condition. Overall mean scores represent the average across the three experts.

**Table 3:** Overall inter-expert reliability of expert evaluations.

Measure	ICC Model	ICC	95% CI
Total score	ICC (2,3)	0.139	−0.350–0.482

ICC, intraclass correlation coefficient; Overall inter-expert reliability was assessed using a two-way random-effects intraclass correlation coefficient with absolute agreement based on average measures across three experts (ICC (2,3)). Negative ICC values indicate agreement lower than expected by chance.

**Table 4:** Expert-specific internal consistency of evaluation items (Cronbach’s α).

Expert	Number of Cases (N)	Number of Items	Cronbach’s α
Expert 1	45	10	0.923
Expert 2	45	10	0.943
Expert 3	45	10	0.923

Cronbach’s α was calculated separately for each expert based on ratings of 45 AI-generated exercise plans across 10 evaluation items.

**Table 5:** Item-level inter-expert reliability across evaluation items (ICC).

Item	Domain	ICC (2,3)	95% CI
Safety	Safety	0.201	−0.142–0.485
Guideline Alignment	Guideline	−0.358	−1.230–0.209
Feasibility	Feasibility	0.020	−0.501–0.401
Personalization	Personalization	0.015	−0.479–0.389
Specificity (FITT-VP)	Prescription	−0.432	−1.237–0.136
Consistency	Quality	−0.005	−0.654–0.416
Clarity	Quality	0.384	0.015–0.635
Completeness	Quality	0.237	−0.224–0.548
Detail Reflection	Quality	0.236	−0.145–0.525
Reproducibility	Quality	0.152	−0.311–0.485

ICC, intraclass correlation coefficient; ICC values were calculated using a two-way random-effects model with absolute agreement based on average measures across three experts (ICC (2,3)). Negative ICC values indicate agreement lower than expected by chance.

**Table 6:** Item-level mean scores by prompt specificity levels.

Item	Stage 1 (Minimal)	Stage 2 (Guideline-Based)	Stage 3 (Structured Schema)
Safety	3.69 ± 0.76	4.07 ± 0.81	3.69 ± 0.85
Guideline Alignment	3.80 ± 0.50	4.16 ± 0.56	3.98 ± 0.69
Feasibility	3.71 ± 0.63	3.78 ± 0.67	3.60 ± 0.75
Personalization	3.38 ± 0.58	3.49 ± 0.76	3.47 ± 0.81
Specificity (FITT-VP)	3.38 ± 0.61	3.42 ± 0.62	3.56 ± 0.99
Consistency	3.49 ± 0.59	3.64 ± 0.53	3.64 ± 0.80
Clarity	3.51 ± 0.66	3.76 ± 0.61	3.78 ± 0.64
Completeness	3.58 ± 0.66	3.73 ± 0.65	3.93 ± 0.86
Detail Reflection	3.00 ± 0.83	3.42 ± 0.69	3.29 ± 0.89
Reproducibility	3.33 ± 0.67	3.56 ± 0.69	3.42 ± 0.89

FITT-VP: frequency, intensity, time, type, volume, progression. Values are presented as mean ± standard deviation based on averaged expert ratings.

## Data Availability

The data supporting the findings of this study, including AI-generated exercise plans and expert evaluation scores, are available from the corresponding author upon reasonable request.

## Supplementary Materials

The following supporting information can be downloaded at https://www.mdpi.com/article/10.3390/jcm15062457/s1, Supplementary Material S1: Standardized Prompt Templates Used for AI-Generated Exercise Prescriptions; Supplementary Material S2: Representative Examples of AI-Generated Exercise Prescriptions Across Prompt Stages; Supplementary Material S3: Expert Evaluation Rubric for AI-Generated Exercise Prescriptions; Supplementary Material S4: Thematic Analysis of Expert Qualitative Feedback on AI-Generated Exercise Prescriptions; Supplementary Material S5: Median and interquartile range (IQR) of expert evaluation scores for each rubric item across prompt stages.
