# Supplementary material for: AI-Generated Exercise Prescriptions for At-Risk Populations: Safety and Feasibility of a Large Language Model Assessed by Expert Evaluation
Source: J Clin Med. 2026 Mar 23;15(6):2457. doi: 10.3390/jcm15062457 (PMC13026971; doi:10.3390/jcm15062457)
Supplement: Supplementary file 1 [file jcm-15-02457-s001.zip › jcm-4195791-supplementary.pdf]

Supplementary Material S1. Standardized Prompt Templates Used for AI-Generated Exercise Prescriptions

1. Overview of Prompt Framework

This supplementary document presents the standardized prompt templates and clinical case input texts used to generate AI-based exercise prescriptions in the present study. All fictional clinical cases were processed using an identical three-stage prompt framework (Prompt 1–3). The overall prompt architecture remained consistent across cases to ensure methodological comparability. Only condition-specific clinical details and relevant guideline references were adapted according to each case.

All prompts were written in English and entered exactly as presented below without additional system-level instructions.

2. Clinical Case Input Texts

**Case 1. Type 2 Diabetes Mellitus + Obesity**
**Participant Profile**<br><br>Male, 55 years old, 7-year history of type 2 diabetes mellitus (on metformin + sulfonylurea), BMI 31.2.<br>Limited exercise experience.<br>Comorbidities: mild peripheral neuropathy, no diabetic retinopathy.<br>Goals: weight reduction and improved glycemic control.
**Case 2. Knee Osteoarthritis + Fall Risk**
**Participant Profile**<br><br>Female, 70 years old, 5-year diagnosis of knee osteoarthritis, BMI 28.<br>Able to walk but experiences knee pain; one prior fall incident.<br>No other comorbidities.<br>Goal: pain reduction, maintenance of walking ability, and fall prevention.

Case 3. Post-Colon Cancer Surgery Recovery
**Participant Profile**<br><br>Male, 60 years old, 6 months post-colon cancer surgery, completed chemotherapy 2 months ago. Deconditioned; currently able to walk for about 15 minutes.<br>No other comorbidities.<br>Goal: physical recovery, fatigue reduction, and improvement of lifestyle habits.<br>Prompt 2 – Guideline-Based

3. Standardized Prompt Templates

Prompt	Description<br><br>(Common Format Used Across All Clinical Cases)
Prompt 1:<br><br>Minimal Information	(1) Instruction<br><br>“Please design a 12-week exercise program for [CLINICAL CASE]. Include weekly frequency, session duration, and intensity.”
Prompt 2:<br><br>Guideline-Based	(1) Instruction<br><br>“Based on [CLINICAL CASE], please develop a 12-week exercise program. Ensure that the plan adheres to international exercise guidelines (e.g., ACSM Guidelines for Exercise Testing and Prescription; ADA Standards of Care; OARSI Guidelines; ACSM Guidelines for Cancer Survivors), and clearly indicate contraindications and precautions. Include aerobic, resistance, balance, and flexibility exercises (if applicable), and specify exercise intensity using RPE or heart rate–based methods.”

Prompt 3:<br><br>Structured Schema<br><br>(Four-Component Format)	(1) Instruction<br><br>"Design a 12-week individualized exercise plan for [CLINICAL CASE]."<br><br>(2) Context<br><br>"Consider the clinical profile characteristics and ensure safety and effectiveness based on relevant clinical and exercise guidelines. Condition-specific contraindications and precautions should be incorporated."<br><br>(3) Input Data<br><br>"Profile for [CLINICAL CASE]: age, sex, condition(s), baseline physical activity level, and exercise-related goals. Specify exercise type, weekly frequency, session duration, methods (e.g., continuous or interval; sets/repetitions), and intensity indicators (RPE, HR, or %1RM)."<br><br>(4) Output Indicator<br><br>"Present the output using the following structure: Week 1–12 exercise table; Safety Box (contraindications, precautions, self-monitoring items; glucose, blood pressure, pain, fall risk, etc.); Guideline Box (summary of applied exercise guidelines; e.g., ACSM Guidelines for Exercise Testing and Prescription; ADA Standards of Care; OARSI Guidelines; ACSM Guidelines for Cancer Survivors); Personalization Box (individual preferences described in the scenario); Feasibility Check (equipment, time, and difficulty suitability)."

Supplementary Material S2. Representative Examples of AI-Generated Exercise Prescriptions Across Prompt Stages

The following representative outputs illustrate AI-generated exercise prescriptions produced under Prompt Stages 1–3 for one fictional clinical case (Type 2 Diabetes Mellitus with Obesity). The presented example reflects structural patterns consistently observed across all evaluated cases. Outputs are presented as generated by the AI model, with only minor formatting adjustments (e.g., spacing and bullet alignment) applied for readability. No content, numerical values, or safety thresholds were modified.

Prompt Stage 1 – Minimal Information

Clinical Case: Type 2 Diabetes Mellitus with Obesity

Important Medical and Safety Precautions

Before starting any new exercise program, the participant must consult their healthcare provider (physician, endocrinologist, or certified diabetes educator) for medical clearance and to discuss potential medication adjustments.

- **Hypoglycemia Risk:** The participant is taking a sulfonylurea, which carries a high risk of hypoglycemia when combined with exercise. Blood glucose levels should be monitored before and after exercise, especially during the initial period. A fast-acting carbohydrate (e.g., glucose tablets or juice) should be carried during sessions. A carbohydrate snack may be necessary if blood glucose is low before exercise.
- **Peripheral Neuropathy:** Mild peripheral neuropathy requires careful selection of activities. Low-impact, non-weight-bearing exercises are preferred to reduce the risk of foot injury and ulceration.
- **Hydration:** Adequate hydration should be maintained before, during, and after exercise.

12-Week Combined Exercise Program Overview

Component	Frequency	Duration	Intensity
Aerobic Training	3–5 days per week	20–45 minutes	Light to Moderate
Resistance Training	2 non-consecutive days per week	20–30 minutes	Light to Moderate
Flexibility/Balance	Daily (warm-up/cool-down)	5–10 minutes	Light

Phase 1: Weeks 1–4 (Foundational / Adaptation)

Focus: Establishing routine, mastering technique, and building initial endurance with low-impact activity.

Aerobic Training (3 days/week)

- Duration: 20–30 minutes per session (minimum 10-minute bouts).
- Intensity: Light to low-moderate; conversation should be easily maintained.
- Activities: Brisk walking or water walking/aqua aerobics (joint-friendly due to BMI and neuropathy).

- Sample Session: 5-minute warm-up walk → 15–20 minutes brisk walking → 5-minute cool-down/stretch.

Resistance Training (2 non-consecutive days/week)

- Duration: 20 minutes per session.
- Intensity: Light; use bodyweight or light resistance bands/dumbbells with controlled movement.
- Target major muscle groups (legs, chest, back, core).
- Sets/Repetitions: 1–2 sets of 10–15 repetitions per exercise.
- Example exercises: Chair squats, wall push-ups, seated dumbbell bicep curls, calf raises (with support).

Flexibility and Balance (Daily)

- Gentle stretching (e.g., seated hamstring and shoulder stretches).
- Simple balance exercises (e.g., single-leg stance with support).

Phase 2: Weeks 5–8 (Progression / Increased Volume)

Focus: Increasing aerobic duration and adding volume to resistance training.

Aerobic Training (4 days/week)

- Duration: 30–40 minutes per session.
- Intensity: Moderate; breathing increases but conversation remains possible.
- Activities: Continue brisk walking or aqua aerobics; introduce cycling (stationary or outdoor).
- Progression: Optional interval walking (e.g., alternating 3 minutes faster pace with 3 minutes slower pace).

Resistance Training (2 non-consecutive days/week)

- Duration: 25 minutes per session.
- Intensity: Moderate; increase resistance so final repetitions are challenging.
- Sets/Repetitions: 2 sets of 10–12 repetitions per exercise.
- Additional exercises: Step-ups (low step), resistance band rows.

Flexibility and Balance (Daily)

- Introduce basic Tai Chi or yoga poses to improve coordination and stability.

Phase 3: Weeks 9–12 (Maintenance and Intensity Refinement)

Focus: Achieving ≥150 minutes of moderate aerobic activity per week and refining resistance intensity.

Aerobic Training (5 days/week)

- Duration: 40–45 minutes per session (total 150–225 minutes/week).
- Intensity: Moderate.
- Continue preferred aerobic activities.

Resistance Training (2 non-consecutive days/week)

- Duration: 30 minutes per session.
- Intensity: Moderate; increase sets.
- Sets/Repetitions: 3 sets of 8–12 repetitions per exercise.
- Consider supervised instruction from a qualified exercise specialist when progressing to machines or free weights.

Lifestyle Integration

- Break up prolonged sitting every 20–30 minutes.
- Increase daily incidental activity (e.g., parking farther away, taking stairs).

This program represents a general template. Individual progress may require adjustments in frequency, duration, or intensity.

Prompt Stage 2 – Guideline-Based

Clinical Case: Type 2 Diabetes Mellitus with Obesity

Overview

The following 12-week exercise program is designed for a 55-year-old male with Type 2 Diabetes Mellitus and Obesity (BMI 31.2), mild peripheral neuropathy, and limited exercise experience, with goals of weight reduction and improved glycemic control.
The program adheres to recommendations from the American College of Sports Medicine (ACSM) and the American Diabetes Association (ADA), emphasizing gradual progression, combined aerobic and resistance training, and condition-specific safety precautions.

Contraindications and Precautions

Given the participant's profile (Type 2 Diabetes treated with sulfonylurea and metformin, obesity, limited experience, mild peripheral neuropathy), the following precautions are emphasized:

Safety Precautions

Hypoglycemia Risk (Sulfonylurea use)

- Blood glucose should be monitored before, and potentially during and after, new or intense exercise sessions.
- If blood glucose is <5.6 mmol/L (<100 mg/dL), a 15 g carbohydrate snack should be consumed before exercise.
- Monitor for delayed hypoglycemia for up to 24 hours post-exercise. Medication adjustment may be required as insulin sensitivity improves.

Hyperglycemia/Ketosis

- Avoid exercise if blood glucose is >16.7 mmol/L (>300 mg/dL) with ketones present.
- If blood glucose is >13.9 mmol/L (>250 mg/dL) without ketones, initiate mild-to-moderate intensity exercise cautiously and monitor closely.

Peripheral Neuropathy Precautions

- Properly fitted, supportive athletic shoes are required.
- Feet should be inspected daily, particularly after exercise.
- High-impact, weight-bearing activities should be avoided.
- Low-impact options (walking, cycling, water-based activity) are preferred.

General Health

- Exercise should be stopped immediately if chest pain, dizziness, irregular heartbeat, or severe pain occurs.

Absolute Contraindications

- Moderate-to-large ketone presence in blood or urine.
- Recent or current hypoglycemic event requiring assistance.
- Symptomatic coronary artery disease.

12-Week Exercise Program Overview

The program follows a three-phase progression model to ensure safety and sustainability. Intensity is primarily guided by the Rating of Perceived Exertion (RPE 6–20 scale).

Component	Weeks 1–4 (Initiation)	Weeks 5–8 (Progression)	Weeks 9–12 (Maintenance)
**Aerobic**	3–5 days/week	5–7 days/week	5–7 days/week
	10–20 min/day (accumulated)	25–40 min/day	≥40 min/day (≥210 min/week)
	RPE 9–11	RPE 11–13	RPE 12–14
	Low-impact modes	Low-impact modes	Brisk walking/cycling
**Resistance**	2 days/week	2–3 days/week	2–3 days/week
	1 set × 10–15 reps	2 sets × 10–15 reps	2–3 sets × 8–12 reps
	RPE 9–11	RPE 11–13	RPE 12–14
**Flexibility**	≥2 days/week	≥3 days/week	≥3 days/week
**Balance**	2 days/week	2–3 days/week	2–3 days/week

Detailed Exercise Components

1. Aerobic Exercise

Goal: ≥150 minutes/week moderate intensity, progressing toward ≥210 minutes/week for weight reduction.

- Mode: Brisk walking, stationary cycling, elliptical, water aerobics.
- Initial intensity: RPE 9–11.
- Progression: Increase duration and frequency before increasing intensity.
- Sedentary interruption strategy: Short activity bouts every 30 minutes.

2. Resistance Exercise

Goal: Improve insulin sensitivity and body composition.

- Mode: Machines, elastic bands, light free weights.
- Target major muscle groups.
- Initial: 1 set × 10–15 repetitions at RPE 9–11.
- Progression: 2–3 sets × 8–12 repetitions at RPE 12–14.
- Avoid Valsalva maneuver during lifts.

3. Flexibility and Balance

Exercise Type	Frequency	Duration/Sets	Rationale
Flexibility	≥2–3 days/week	10–30 sec, 2–4 reps	Maintain range of motion
Balance	≥2–3 days/week	1–2 sets, 30–60 sec	Address neuropathy-related balance issues

Examples: Supported single-leg stance, heel-to-toe walking.

Guideline Alignment (Weeks 9–12 Target)

Category	ACSM/ADA Recommendation	Program Adherence
Aerobic Frequency	≥3–7 days/week	5–7 days/week

Aerobic Duration	≥150 min/week	≥210 min/week
Aerobic Intensity	Moderate–Vigorous	RPE 12–14
Resistance	≥2–3 days/week	2–3 days/week
Combined Training	Aerobic + Resistance	Included
Neuropathy Consideration	Low-impact, footwear	Incorporated

Prompt Stage 3 – Structured Schema

Clinical Case: Type 2 Diabetes Mellitus with Obesity

12-Week Individualized Exercise Plan

This 12-week individualized exercise plan is designed for a participant with Type 2 Diabetes Mellitus (T2DM) and obesity, focusing on weight reduction, improved glycemic control, enhanced cardiorespiratory fitness, and muscular strength/endurance.

Week 1–12 Exercise Prescription Table

Exercise Component	Weekly Frequency	Session Duration	Method / Sets / Reps	Intensity	Progression Rule
**Aerobic Exercise**	5–7 days/week	Weeks 1–4: 20–30 min<br>Weeks 5–8: 30–40 min<br>Weeks 9–12: 40–50 min	Continuous	RPE 11–12 → RPE 13<br>50–70% HRR	Increase duration by 5–10 min every 2 weeks. Increase intensity after Week 4 if well tolerated.
**Aerobic Type**	Walking, stationary cycling, water aerobics (low-impact)				Introduce interval training (2 min moderate / 1 min light) twice per week after Week 6.
**Resistance Exercise**	2–3 non-consecutive days/week	Weeks 1–4: 20–30 min<br>Weeks 5–12: 30–45 min	Weeks 1–4: 1–2 sets × 10–15 reps<br>Weeks 5–12: 2–3 sets × 8–12 reps	RPE 13–15	Gradually increase sets and load after Week 4; prioritize correct form.
**Resistance Type**	Major muscle groups (e.g., sit-to-stand, wall push-ups, modified planks, resistance band rows/presses)				Introduce step-ups, dumbbell curls, or light machines after Week 6 if tolerated.
**Flexibility/Balance**	Daily (post-exercise), 1–2×/day	5–10 min	Static stretches 15–30 sec; balance 10–15 reps/side	Light tension, no pain	Increase hold time and introduce single-leg standing after Week 4.

Heart Rate Reserve (HRR) Calculation
Target HR = [(HRmax − HRrest) × %Intensity] + HRrest
Estimated HRmax = 220 − 55 = 165 bpm (Karvonen formula recommended for precision).

Safety Box: Contraindications, Precautions, and Self-Monitoring

Component	Detail
**Absolute Contraindications**	Unstable angina; uncontrolled BP >200/110 mmHg; significant retinopathy; uncontrolled blood glucose >300 mg/dL; presence of ketones.
**Precautions**	Monitor glucose before/after exercise due to sulfonylurea use; carry fast-acting carbohydrates; use low-impact modes; inspect feet daily; avoid high-impact activities; follow "start low and go slow" approach.
**Self-Monitoring**	Stop exercise if BG <70 mg/dL or >250 mg/dL with ketones; stop if pain >3/10; perform balance exercises near support; use RPE 11–15 scale; maintain hydration.

Guideline Box: Summary of Applied ACSM/ADA Recommendations

Guideline Aspect	Application	Rationale
Aerobic Exercise	≥150 min/week moderate (progressing toward ≥210 min/week)	Improves glycemic control and supports weight loss
Resistance Exercise	2–3 non-consecutive days/week, all major muscle groups	Enhances insulin sensitivity and preserves lean mass
Exercise Timing	Interrupt prolonged sitting with short activity bouts	Improves postprandial glucose control
Neuropathy/Safety	Low-impact exercise; foot inspection; glucose monitoring	Reduces injury and metabolic risk

Personalization Box

Factor	Consideration	Plan Integration
Exercise Experience	Limited; potential gym intimidation	Begin with walking and home-based resistance exercises
Environment	Access to parks/sidewalks/home setting	Outdoor walking; in-home resistance; optional gym in Phase 3
Motivation	High dropout risk	Encourage social support and small weekly goals

Feasibility Check

Factor	Suitability	Alternatives
Equipment	Requires shoes, glucose meter, resistance bands	Household items as resistance
Time	40–90 min/day (can be split)	Two 15–25 min sessions if needed
Difficulty	Low initial RPE 11–12 with progression	Modify exercises if too easy/difficult
Environment	Weather-dependent outdoor walking	Indoor cycling, mall walking, step-ups

Supplementary Material S3. Expert Evaluation Rubric for AI-Generated Exercise Prescriptions

Evaluation Item	Description	Scale (1–5)
		1=Very poor – 5=Excellent
Safety	Presentation of contraindications and precautions (including specific values for blood glucose, blood pressure, etc.)	
Guideline adherence	Degree of compliance with international exercise guidelines (frequency, intensity, cut-offs, etc.)	
Feasibility	Practical applicability considering equipment, environment, and patient's living context	
Personalization	Reflection of scenario-specific characteristics (age, disease, medication, environment)	
Specificity (FITT-VP)	Inclusion of FITT-VP elements (frequency, intensity, time, type, volume, progression)	
Consistency	Progressive structure of the 12-week program and internal coherence	
Clarity	Ease of understanding for patients (clarity, limited jargon use)	
Completeness	Balance and comprehensiveness across aerobic, resistance, balance, and flexibility components	
Detail Reflection	Reflection of detailed conditions from guidelines (e.g., blood glucose cut-offs, Valsalva maneuver restriction)	
Reproducibility	Reproducibility of results when generated multiple times under identical conditions	

Reviewer signature: ___________________________ Date: _____________

Supplementary Material S4. Thematic Analysis of Expert Qualitative Feedback on AI-Generated Exercise Prescriptions

	Key Expert Observations (Synthesis)	Representative Expert Comments (Quotes)
Theme 1: Trade-off between Structure & Specificity	While Stage 3 followed the structured prompt strictly, it often led to "generalized explanations" rather than "actionable prescriptions."	"Structure is faithful, but the content is somewhat generalized, limiting personalization." / "More like an 'explanation of principles' rather than a 12-week prescription."
Theme 2: Ambiguity in Progression Logic	Experts noted a lack of clear numerical criteria for progressing intensity (e.g., when to increase weight or sets) across all stages.	"Lacks clear criteria for increasing resistance (e.g., reps, sets, rest)." / "Weekly prescription strength is weak compared to standard programs."
Theme 3: Subjective Safety Thresholds	Experts held different views on safety: some prioritized "contraindications," while others focused on "alternative exercises." This explains the low ICC.	"Missing criteria for returning to exercise after symptoms (pain/dizziness)." / "Needs clearer support conditions/alternatives for patients with fall risks."
Theme 4: Practical Feasibility & Compliance	AI often missed the nuance of patient psychology (e.g., fear of certain movements) which human experts prioritize for adherence.	"Specific exercise tasks should be suggested to address patient fear and improve compliance (e.g., push-ups as an alternative to bench press)."

Supplementary Material S5. Median and interquartile range (IQR) of expert evaluation scores for each rubric item across prompt stages.

**Item**	**Stage 1 Median (IQR)**	**Stage 2 Median (IQR)**	**Stage 3 Median (IQR)**
Safety	4 (4–5)	4 (3–4)	3 (3–4)
Guideline Alignment	4 (4–4)	4 (4–4)	4 (4–4)
Feasibility	4 (3–4)	4 (3–4)	4 (3–4)
Personalization	3 (3–4)	4 (3–4)	3 (3–4)
Specificity (FITT-VP)	3 (3–4)	4 (3–4)	3 (3–4)
Consistency	4 (3–4)	4 (3–4)	4 (3–4)
Clarity	4 (3–4)	4 (3–4)	4 (3–4)
Completeness	4 (3–4)	4 (4–4)	4 (3–4)
Detail Reflection	3 (3–4)	4 (3–4)	3 (3–3)
Reproducibility	3 (3–4)	4 (3–4)	3 (3–4)

FITT-VP, frequency, intensity, time, type, volume, progression. Values are presented as median (interquartile range).
